# Clinical and Ultrasonic Risk Factors for Lateral Lymph Node Metastasis in Papillary Thyroid Microcarcinoma: A Systematic Review and Meta-Analysis

**DOI:** 10.3389/fonc.2020.00436

**Published:** 2020-04-03

**Authors:** Shuai Xue, Zhe Han, Qiyu Lu, Peisong Wang, Guang Chen

**Affiliations:** Department of Thyroid Surgery, The 1^st^ Hospital of Jilin University, Changchun, China

**Keywords:** risk factor, lateral lymph node metastasis, papillary thyroid microcarcinoma, meta-analysis, review

## Abstract

**Background:** Clinical and ultrasonic risk factors for lateral lymph node metastasis (LLNM) in papillary thyroid microcarcinoma (PTMC) are not well-defined. Herein, a systematic review and meta-analysis was conducted to investigate clinicopathologic and ultrasonic risk features for LLNM in PTMC.

**Methods:** A systematic search of electronic databases (PubMed, Embase, Cochrane Library, and Web of Science) for studies published until April 2019 was performed. Case–control studies and randomized controlled trials that studied clinical and ultrasonic risk factors of LLNM in PTMC were included.

**Results:** Fourteen studies (all retrospective studies) involving 43,750 patients met final inclusion criteria. From the pooled analyses, younger age<45 (OR, 1.55; 95% CI, 1.16–2.07; *P* = 0.003), male patients (OR, 1.94; 95% CI, 1.55–2.42; *P* < 0.00), extrathyroidal extension (OR, 3.63; 95% CI, 2.28–5.77; *P* <0.00), tumor multifocality (OR, 2.24; 95% CI, 1.53–3.28; *P* <0.00), tumor > 0.5 cm (OR, 2.24; 95% CI, 1.53–3.28; *P* < 0.00), central lymph node metastasis (OR, 5.61; 95% CI, 4.64–6.79; *P* < 0.00), >25% tumor contact with thyroid capsule (OR, 6.66; 95% CI, 1.96–22.65; *P* = 0.002), tumor calcification (OR, 2.90; 95% CI, 1.71–4.93; *P* < 0.00), upper tumor (OR, 3.18; 95% CI, 2.23–4.55; *P* < 0.00) were significantly associated with increased risk of LLNM in PTMC, while Hashimoto's thyroiditis and other ultrasonic features (solid tumor, hypoechoic tumor, smooth margin, and taller than wide tumor) were not significantly associated with LLNM in PTMC.

**Conclusions:** Our analysis identified several clinicopathologic and ultrasonic factors associated with LLNM in PTMC. This finding highlights the need for a cautious and frequent postoperative surveillance of the lateral neck in high-risk PTMC patients. Moreover, high-risk ultrasonic features also need to be considered during selection of PTMC for active surveillance.

## Introduction

The global incidence of papillary thyroid carcinoma (PTC) has increased substantially during past decade, with 4.4% annual percent increase in the United States from 1974 to 2013 (1–3). This has been driven largely by the rise in papillary thyroid microcarcinoma (PTMC), which is defined as PTC measuring ≤1 cm in greatest dimension (2, 3). Although the majority of PTMCs are indolent with <0.5% thyroid-cancer related death, some (1–5%) may have locoregional recurrence, which is still a major concern for patients and clinicians (4). Mounting studies reported that lateral lymph node metastasis (LLNM) was associated with locoregional recurrence for PTMC (4–6). The revised American Thyroid Association (ATA) guidelines in 2015 also considered around 20% PTC patients with LLNM would have structural recurrence in the future (7).

It is well-accepted that lateral neck dissection (LND) is only recommended for PTMC patients when LLNM is diagnosed by preoperative evaluation such as physical examination, radiological imaging, and/or fine needle aspiration (FNA) (7). Prophylactic LND is not recommended because no reliable clinicopathological features are identified which can differentiate the subset of high-risk PTMC, which are more likely accompanied with microscopic lymph nodes and the potential of progressing to clinical LLNM. But some researchers still believed early treatment for PTMC with LLNM might be appropriate as long as high-risk PTMC patients could be distinguished by clinical and/or ultrasonic risk factors (8).

Moreover, active surveillance could be recommended as the first-line treatment for low-risk PTC patients (7). But around 3.8% patients under active surveillance will have novel LLNM, which is also a major concern for patients and clinicians (9, 10). So it is necessary to identify patients at high-risk for LLNM, which helps the enrollment of low-risk PTMC in active surveillance protocols (9, 10).

Accordingly, several studies have published on the preoperative clinicopathologic risk factors of LLNM for PTMC (11, 12). However, there have been no consensus on this, and the subject remains debatable. Considering the low incidence of LLNM in PTMC, a single study with small patient number may draw an unreliable conclusion with a considerable bias. Therefore, we performed a systematic review to evaluate the clinicopathologic and ultrasonic predictive factors for LLNM in PTMC.

## Methods

According to the guidelines proposed by the preferred reporting items for systematic reviews and meta-analyses (PRISMA in Supplementary Material) statement, we conducted this systematic review and meta-analysis.

### Search Strategy

A systematic search of PubMed, Embase, Cochrane Library, and Web of Science was conducted for articles published until April 2019. The following terms were used in searching: “lateral lymph node metastasis” or “lateral lymph node dissection” combined with “papillary thyroid microcarcinoma” with language restriction “English.” All possible spelling and synonyms were also used for searching. Details of the search strategy are provided as shown in Supplementary File 1. The title, abstract or descriptors was reviewed independently by two authors (SX and PSW) to identify related studies for extensive review.

### Study Selection

Studies returned from the search were checked by the following inclusion criteria: (1) original articles; (2) PTMC patients who received thyroidectomy as primary surgical procedure; (3) evaluation of clinicopathologic and/or ultrasonic risk factors for LLNM; and (4) study of the association between LLNM and relevant risk factors. Initially, titles, and abstracts were checked to include studies which fulfilled the inclusion criteria. After excluding studies which did not fulfill inclusion criteria, letters to the editor, abstracts, and meeting posters were also excluded. The same reviewers independently assessed eligibility after obtaining full text of candidate studies. In case of disagreement, a third investigator (ZH) was consulted. Discrepancies were resolved by discussion and consensus.

### Data Extraction

Two investigators (SX and PSW) independently summarized the studies meeting the inclusion criteria and performed data extraction. Disagreement was resolved by discussion and a third investigator (ZH) was consulted. We extracted the following data for each study: first author's last name, publication year, country, study design, sample size, patient characteristics (age and gender), surgical intervention (surgery type and therapeutic LND scope), and percentage of patients with LLNM. Following review by an expert panel (ZH and GC), we selected 14 factors that had been analyzed in at least three studies. These factors included clinicopathological factors like age, sex, extrathyroidal extension, multifocality, tumor size, Hashimoto's thyroiditis (HT), central lymph node metastases (CLNM), and ultrasonic characteristics of tumor like >25% contact with thyroid capsule, calcification, composition, echo, margin, shape, location.

### Risk of Bias Analysis

The Risk of Bias Assessment Tool for Non-randomized Studies (RoBANS) was used in our study to evaluate the quality of non-randomized experimental studies. The six domains included in the RoBANS tool are the selection of participants, confounding variables, measurement of intervention, blinding for outcome assessment, incomplete outcome data, and selective outcome reporting were considered for assessment. Two reviewers (ZH and QYL) independently reviewed each term for every study, and disagreement was resolved by re-evaluation and a third investigator (GC) was consulted. The measurement of each item is categorized as low risk, high risk, or unclear risk. Review Manager 5.3 (Cochrane Collaboration, Oxford, UK) was used to report the result of this evaluation.

### Statistical Analyses

Revman software (version 5.3; Cochrane Collaboration, Oxford, UK) and Stata software (version 12.0; Stata Corporation, College Station, TX, USA) were used for this meta and statistical analysis. We also calculated the odds ratios (ORs) and 95% confidence intervals (CIs) for estimating the association between binary factors and LLNM. Meta-analysis was performed using the random-effects model or the fixed-effect model according to the absence or presence of significant heterogeneity. The heterogeneity among studies was evaluated by Cochran's chi-squared statistics and with significance set at *P* < 0.10. The *I*^2^ statistic was used to quantify heterogeneity as followings: (1) exclude heterogeneity if *I*^2^ was from 0 to 40%; (2) moderate heterogeneity if *I*^2^ was from 30 to 60%. (3) Substantial heterogeneity if *I*^2^ was from 50 to 90%; (4) considerable heterogeneity if *I*^2^ was from 75 to 100%. If heterogeneity was present among the included studies, sensitivity analysis were performed to explore the origins of the heterogeneity.

The risk of publication bias was analyzed using Egger's test and was presented as a funnel plot. The statistical power of Egger's test was mainly dependent on the number of studies included in a meta-analysis. The *P* < 0.1 has been used as evidence of asymmetry for funnel plot because of limited number of studies in this study.

## Results

### Description of Studies

**Figure 1:**
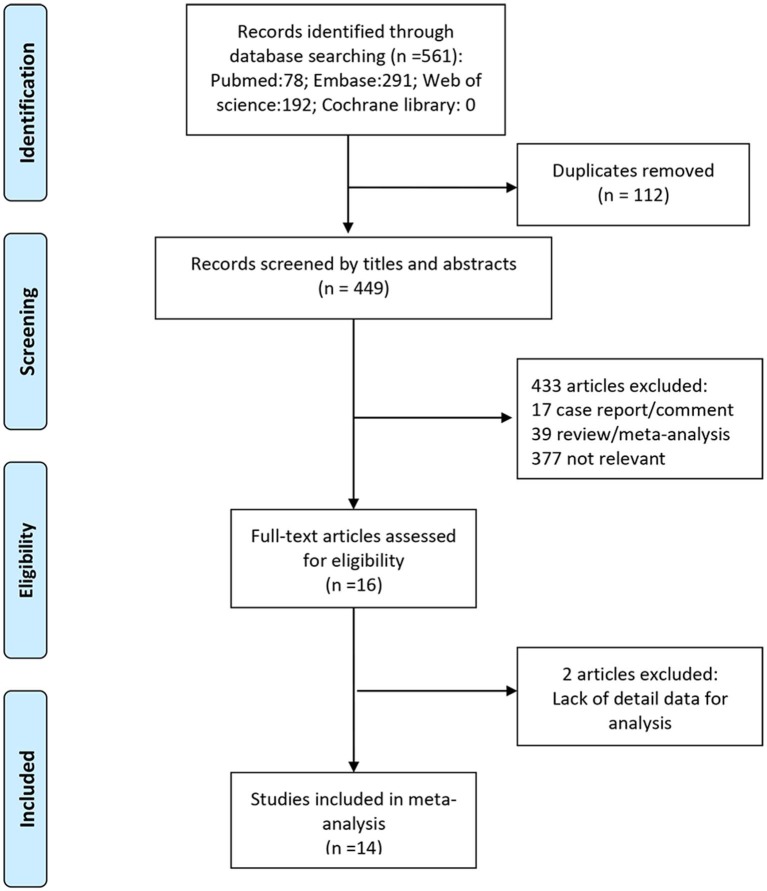
Preferred reporting items for systematic reviews and meta-analyses flow chart of the study selection process, showing the number of studies excluded at each step and the reasons for exclusion from the systematic review and meta-analysis.

We identified a total of 561 studies during initial literature search. After evaluation of the titles and abstracts, 112 duplicate studies and another 433 studies were excluded. After scrutiny of the full text of the remaining 16 articles, a further 2 studies were excluded for various reasons as shown in Figure 1. Finally, 14 studies (with a total of 43,750 patients) were included in this meta-analysis, which all were retrospective studies (4, 13–25). Figure 1 and Table 1 show the study selection process and the characteristics of the included studies, respectively.

**Table 1:** Characteristics of selected studies.

	**References**	**Country**	**Study design**	**Sample size**	**Mean age, y**	**Male, (%)**	**Surgery**	**Therapeutic LLND**	**LLNM, N (%)**
1	Kwak et al. (13)	SK	RS	671	48	77 (11.5)	TT/LT or CLND	Radical (II–V)	25 (3.7)
2	Lin et al. (14)	China	RS	61	49.2	9 (14.8)	TT and CLND and LLND	Radical (II–V)	17 (28)
3	Kim et al. (15)	SK	RS	490	45.9	62 (12.7)	TT and CLND	Radical (II–V)	5 (8.1)
4	Zeng et al. (16)	China	RS	176	44.9	45 (25.6)	TT and CLND and LLND	NA	62 (35.2)
5	Shin et al. (17)	SK	RS	588	45.2	93 (15.8)	TT/LT and CLND	NA	26 (4.4)
6	Lin et al. (18)	China	RS	31,017	50.9	5,725 (18.5)	TT/LT or CLND	NA	1,684 (5.4)
7	Siddiqui et al. (4)	USA	RS	273	49	41 (15)	TT/LT or CLND	Radical (I–V)	18 (6.5)
8	Kim et al. (19)	SK	RS	5,656	48	1,002 (17.7)	TT/LT or CLND	Selective or radical	518 (9.1)
9	Jeon et al. (20)	SK	RS	395	48.5	76 (19.2)	TT and CLND and LLND	NA	196 (49.6)
10	Xu et al. (21)	China	RS	3,607	47.5	868 (24.1)	TT/LT and CLND	Selective (II–IV)	38 (1.1)
11	Wang et al. (22)	China	RS	169	46	49 (29)	TT and CLND	Selective (II–IV)	18(10.7)
12	Tao et al. (23)	China	RS	66	43.5	13 (19.7)	TT/LT and CLND	Radical (II–V)	6 (9.1)
13	Liu et al. (24)	China	RS	366	41	103 (28.1)	TT/LT and CLND	Radical (II–V)	62 (16.9)
14	Zhao et al. (25)	China	RS	215	42	75 (34.9)	TT and CLND and LLND	Radical (II–V)	163 (75.8)

*LLNM, lateral lymph node metastasis; SK, South Korea; RS, retrospective study; TT, total thyroidectomy; LT, lebectomy; CLND, central lymph node dissection; LLND, lateral lymph node dissection; NA, not available*.

#### Age and LLNM

**Figure 2:**
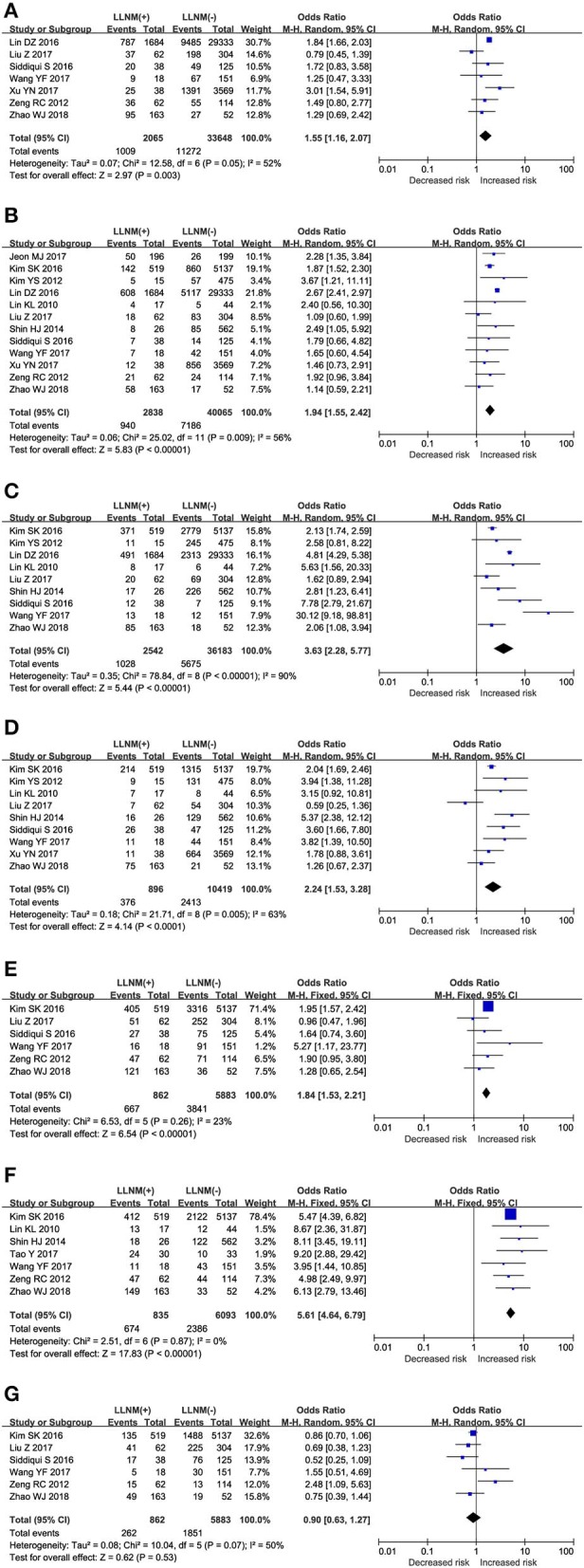
The role of clinical risk factors for LLNM in PTMC. Forest plots for the effects of **(A)** Age. **(B)** Sex. **(C)** ETE. **(D)** Multifocal. **(E)** Tumor size. **(F)** CLNM. **(G)** HT.

The effect of age on the risk of LLNM was investigated in 7 studies (Figure 2A). A random-effects model was used due to the moderate heterogeneity (*P* = 0.05; *I*^2^ = 52%). On pooled analysis, the risk of LLNM was significantly higher in patients with younger age<45 (OR, 1.55; 95% CI, 1.16–2.07; *P* =0.003).

#### Sex and LLNM

The effect of sex on the risk of LLNM was investigated in 12 studies (Figure 2B). A random-effects model was applied due to the moderate heterogeneity (*P* = 0.009; *I*^2^ = 56%). On pooled analysis, the risk of LLNM was significantly higher in male patients (OR, 1.94; 95% CI, 1.55–2.42; *P* < 0.00).

#### ETE and LLNM

The effect of ETE on the risk of LLNM was investigated in 9 studies (Figure 2C). A random-effects model was applied due to the moderate heterogeneity (*P* < 0.00; *I*^2^ = 90%). On pooled analysis, the risk of LLNM was significantly higher in patients with ETE (OR, 3.63; 95% CI, 2.28–5.77; *P* < 0.00).

#### Multifocality and LLNM

The effect of multifocality on the risk of LLNM was investigated in 9 studies (Figure 2D). A random-effects model was applied due to the moderate heterogeneity (*P* = 0.009; *I*^2^ = 63%). On pooled analysis, the risk of LLNM was significantly higher in patients with multifocal tumor (OR, 2.24; 95% CI, 1.53–3.28; *P* < 0.00).

#### Tumor Size and LLNM

Assessment of tumor size as a risk factor for LLNM was conducted in 9 studies (Figure 2E). A fixed-effects model was applied due to low heterogeneity (*P* = 0.37; *I*^2^ = 23%). On pooled analysis, the risk of LLNM was significantly higher in patients with tumor larger than 0.5 cm. (OR, 2.24; 95% CI, 1.53–3.28; *P* < 0.00).

#### CLNM and LLNM

Assessment of CLNM as a risk factor for LLNM was conducted in 7 studies (Figure 2F). A fixed-effects model was applied due to low heterogeneity (*P* = 0.87; *I*^2^ = 0%). On pooled analysis, the risk of LLNM was significantly higher in patients with CLNM (OR, 5.61; 95% CI, 4.64–6.79; *P* < 0.00).

#### HT and LLNM

The effect of HT on the risk of LLNM was investigated in 6 studies (Figure 2G). A random-effects model was applied due to the moderate heterogeneity (*P* = 0.07; *I*^2^ = 50%). On pooled analysis, the risk of LLNM was not significantly lower in HT patients (OR, 0.90; 95% CI, 0.63–1.27; *P* < 0.53).

#### >25% Contact of Tumor With Thyroid Membrane and LLNM

**Figure 3:**
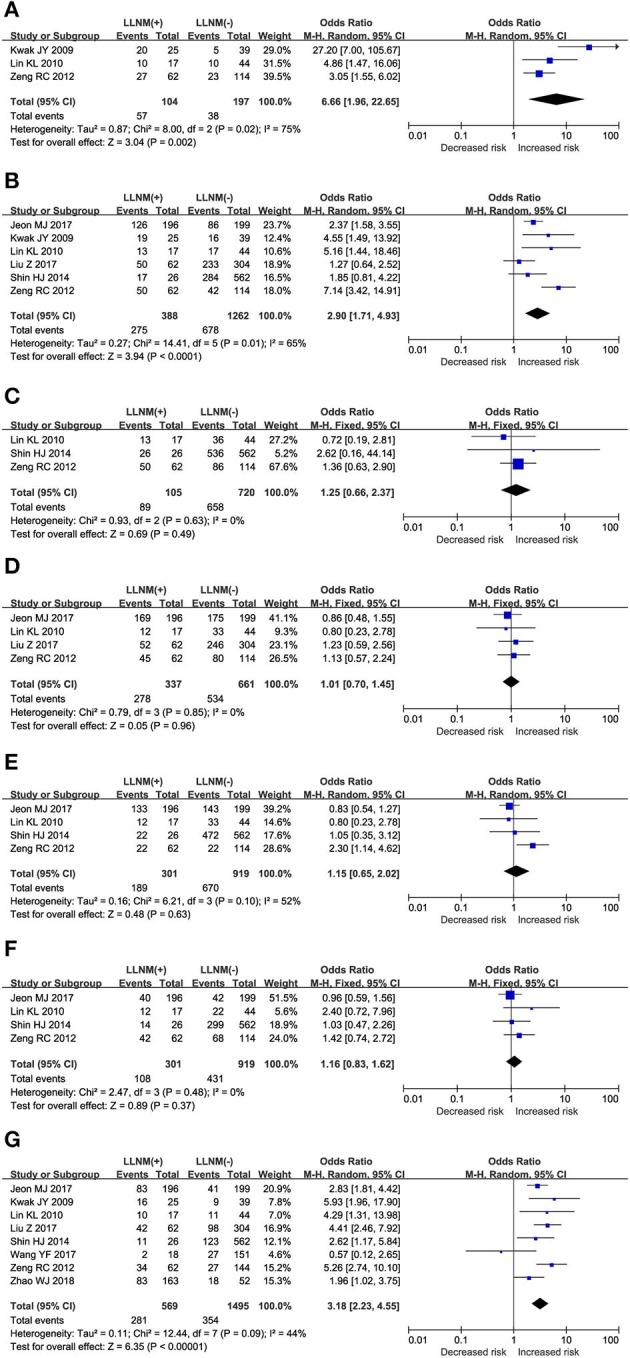
The role of ultrasonic risk factors for LLNM in PTMC. Forest plots for the effects of **(A)** Contact>25% thyroid membrane. **(B)** Calcification. **(C)** Composition. **(D)** Echo. **(E)** Margin. **(F)** Shape. **(G)** Location.

The prediction of >25% contact of tumor with thyroid membrane on the risk of LLNM was investigated in 3 studies (Figure 3A). A random-effects model was applied due to the moderate heterogeneity (*P* = 0.02; *I*^2^ = 75%). On pooled analysis, the risk of LLNM was significantly higher in patients with tumor contact >25% thyroid membrane (OR, 6.66; 95% CI, 1.96–22.65; *P* = 0.002).

#### Calcification and LLNM

The prediction of calcification on the risk of LLNM was investigated in 6 studies (Figure 3B). A random-effects model was applied due to the moderate heterogeneity (*P* = 0.01; *I*^2^ = 65%). On pooled analysis, the risk of LLNM was significantly higher in patients with tumor calcification (OR, 2.90; 95% CI, 1.71–4.93; *P* < 0.00).

#### Composition and LLNM

Assessment of tumor composition as a risk factor for LLNM was conducted in 3 studies (Figure 3C). A fixed-effects model was applied because of low heterogeneity (*P* = 0.63; *I*^2^ = 0%). On pooled analysis, the risk of LLNM was not significantly higher in patients with solid tumor (OR, 1.25; 95% CI, 0.66–2.37; *P* < 0.00).

#### Echo and LLNM

Assessment of tumor echo as a risk factor for LLNM was conducted in 4 studies (Figure 3D). A fixed-effects model was applied because of low heterogeneity (*P* = 0.85; *I*^2^ = 0%). On pooled analysis, the risk of LLNM was not significantly higher in patients with hypoechoic tumor (OR, 1.01; 95% CI, 0.70–1.45; *P* = 0.96).

#### Margin and LLNM

The prediction of tumor margin on the risk of LLNM was investigated in a total of 4 studies (Figure 3E). A random-effects model was applied due to the moderate heterogeneity (*P* = 0.10; *I*^2^ = 52%). On pooled analysis, the risk of LLNM was not significantly higher in patients with smooth margin (OR, 1.15; 95% CI, 0.65–2.02; *P* < 0.63).

#### Shape and LLNM

Assessment of tumor shape as a risk factor for LLNM was conducted in a total of 4 studies (Figure 3F). A fixed-effects model was applied due to low heterogeneity (*P* = 0.48; *I*^2^ = 0%). On pooled analysis, the risk of LLNM was not significantly higher in patients with taller than wide tumor (OR, 1.16; 95% CI, 0.83–1.62; *P* < 0.37).

#### Location and LLNM

The prediction of tumor location on the risk of LLNM was investigated in a total of 8 studies (Figure 3G). A random-effects model was applied due to the moderate heterogeneity (*P* = 0.09; *I*^2^ = 44%). On pooled analysis, the risk of LLNM was significantly higher in patients with upper tumor (OR, 3.18; 95% CI, 2.23–4.55; *P* < 0.00).

### Assessment of Study Quality and Bias

**Figure 4:**
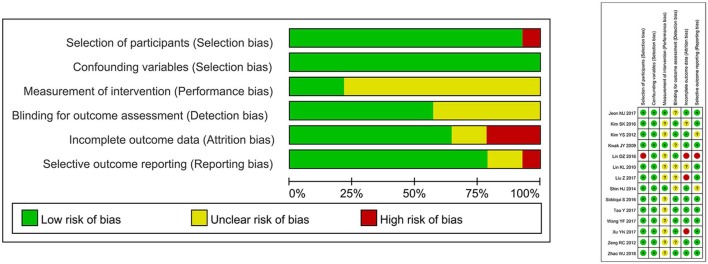
Risk of bias graph: review authors' judgements about each risk of bias item presented as percentages across all included studies.

As shown in Figure 4, the risk for bias are summarized for 14 studies included in this meta-analysis by RoBANS tool. In the 14 studies included in the assessment, 100% were low risk for confounding variables and 92.9% were evaluated as low risk in the selection of participants. The unclear risk ratios of performance and detection biases were estimated to be 78.6 and 42.9%, respectively. The low risk ratios of attrition and reporting biases were 64.3 and 78.6%, respectively.

### Sensitivity Analysis

Overall, leave-one-out meta-analysis revealed that age, sex, multifocal, HT, contact>75%, calcification and margin did not retain significance when single studies were excluded. For ETE and tumor location, no single studies were identified for the huge heterogeneity. The detailed results, including the forest plots, of the sensitivity analyses have been reported in Supplementary File 2.

### Publication Bias

Publication bias was evaluated by Egger test and no bias existed in this meta-analysis, as shown in Supplementary File 3.

## Discussion

To the best of our knowledge, this is the first meta-analysis to assess clinical and ultrasonic risk factors for LLNM in PTMC. It was found that younger age, male gender, ETE, multifocal tumors, larger tumor size, and positive CLNM were clinical risk factors for LLNM in PTMC. Additionally, >25% tumor contact with thyroid capsule, calcification, and upper location were ultrasonic risk features for LLNM. In contrast, HT and other US features (such as composition, echo, margin, shape) were not considered as risk factors of LLNM in these patients.

High heterogeneity with an *I*^2^ >50% was found in the analysis of age, sex, ETE, multifocality, HT, >25% contact with thyroid capsule, calcification as well as margin, and moderate heterogeneity with an *I*^2^ >30% was found when analyzing tumor location. Interestingly, after the removal of one study from the analysis, similar results were confirmed, but the heterogeneity was decreased remarkably in the analysis of age, sex, multifocal, HT, contact>25%, calcification, and margin. Possible reasons for high heterogeneity were presented as Supplementary File 2. In contrast, the *I*^2^ values for the analysis of ETE and tumor location were not changed significantly in the sensitivity analysis.

Younger age was identified to be associated with aggressiveness of PTC, such as vascular invasion and lymph node metastases (26). However, there is mounting evidence that younger PTC patients have a better disease-specific survival although they present high-risk clinical-pathological features (27–29). For PTMC under active surveillance, younger age was also associated with more probability of disease progression like tumor enlargement by ≥3 mm or novel appearance of nodal metastasis (30). The specific mechanism of age-dependent tumorigenesis and tumor progression has not been intensively investigated. Tumor surveillance by the immune system, which varies according to age, may be the reason for the difference in prognosis between younger and older patients (31).

Similarly, cervical lymph node metastases and ETE were more frequent in PTC patients with multifocality (32, 33). A meta-analysis from Australia summarized 21 papers and concluded the multifocality was an independent risk factor for recurrence, tumor progression, and aggressiveness (34). However, Wang et al. retrospectively reviewed 2,638 PTC patients from 11 medical centers from 6 countries and found that tumor multifocality has no independent risk prognostic value in clinical outcomes of PTC (35). This result was also validated on a series of 89,680 patients from SEER database (35). Recently, a study from Israel compared 690 PTC patients using propensity score matching analysis and demonstrated multifocality was not an independent prognostic factor for long-term outcomes of PTC (36). Multifocal PTC can represent either an intraglandular spread from a single primary tumor or multiple independent foci accompanied by intrathyroidal metastasis (37–39). These different clonal origins of multifocal tumor have distinct growth patterns, which may explain discrepancy between the above-discussed studies (40).

Furthermore, another novelty of this study is the first meta-analysis for ultrasonic features of LLNM in PTMC. A >25% tumor contact with thyroid capsule is related with the degree of ETE, which is also associated with LLNM of PTMC. Moreover, microcalcification(s) at US can also predict a diagnosis of malignancy, clinically cervical lymph node metastases, and prognosis, as reported previously (41, 42). In addition, an increasing number of studies have reported that upper tumor location was related with LLNM in PTC and PTMC no matter whether central neck had metastatic lymph node or not (11, 20, 43, 44). The presence of a direct lymphatic drainage from the upper third of thyroid to the neck may explain this finding (44, 45).

## Strengths and Weaknesses

By meta-analyzing populations from different studies, the present study was able to evaluate the risk factors of LLNM for PTMC in a larger study sample and to adjust the results for the presence of some confounding factors. Except for ETE and tumor location, high heterogeneity of other risk factors was compensated by sensitivity analysis. The results of ETE and tumor location should be interpreted with caution because of moderate heterogeneity, and further study is needed to confirm the corresponding results.

This meta-analysis has some potential limitations. First, patients in the 14 studies were predominantly Asian. Whether ethnicity plays a role in LLNM remains unknown. Accordingly, risk factors of LLNM in PTMC from other races need further evaluation. Second, the limited number of studies hindered the implementation of subgroup and meta regression analysis. Third, the retrospective and non-randomized nature of all studies included in the analysis might be considered a source of bias. This provided associative, not causal, evidence, and mandates caution when interpreting these results. In future trials, randomized controlled trials of a higher methodological quality are needed to improve the quality of evidence.

## Conclusion

Taken together, several risk factors for LLNM in PTMC patients which are readily available in clinical settings were identified in our systematic review and meta-analysis. The identification of high-risk patients is of utmost importance to plan a more cautious and frequent evaluation of lateral neck in the post-operative course. Moreover, high-risk ultrasonic features also need to be considered during selection of PTMC for active surveillance.

## Author Contributions

All authors listed have made a substantial, direct and intellectual contribution to the work, and approved it for publication.

### Conflict of Interest

The authors declare that the research was conducted in the absence of any commercial or financial relationships that could be construed as a potential conflict of interest.
