# Supplementary material for: Clinical and Ultrasonic Risk Factors for Lateral Lymph Node Metastasis in Papillary Thyroid Microcarcinoma: A Systematic Review and Meta-Analysis
Source: Front Oncol. 2020 Apr 3;10:436. doi: 10.3389/fonc.2020.00436 (PMC7145902; doi:10.3389/fonc.2020.00436)

Table S3 Egger’s test for evaluating publication bias

| Clinical pathologic features | Egger (p value) |
| --- | --- |
| Age | 0.529 |
| Sex | 0.39 |
| ETE | 0.784 |
| Multifocal | 0.212 |
| Tumor size | 0.663 |
| CLNM | 0.622 |
| HT | 0.744 |
|  |  |
| Ultrasound features |  |
| Contact>25% | 0.305 |
| Calcification | 0.245 |
| Composition | 0.891 |
| Echo | 0.625 |
| Margin | 0.722 |
| Shape | 0.242 |
| Location | 0.102 |

ETE: extrathyroidal extension; CLNM: central lymph node metastasis; HT: Hashimoto’s thyroiditis;

Figure S3 Funnel plots for egger test

A) Age


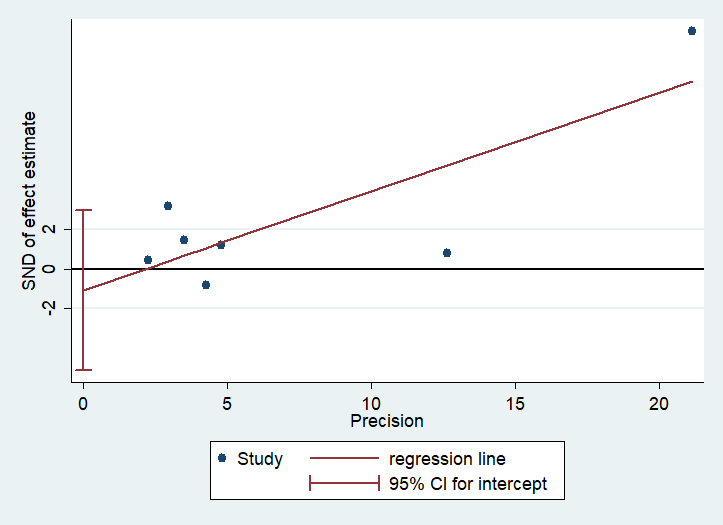


B) Sex


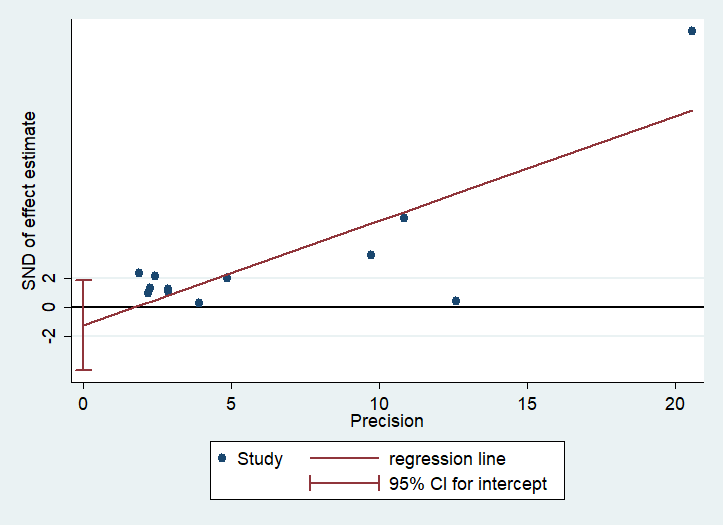


C) ETE


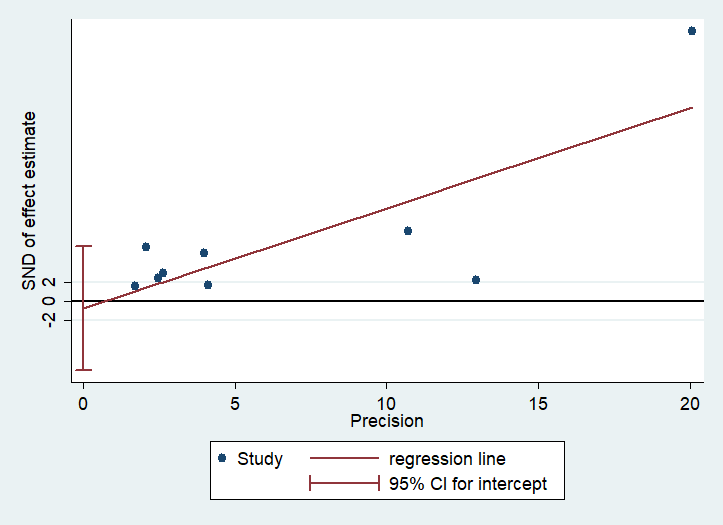


D) Multifocal


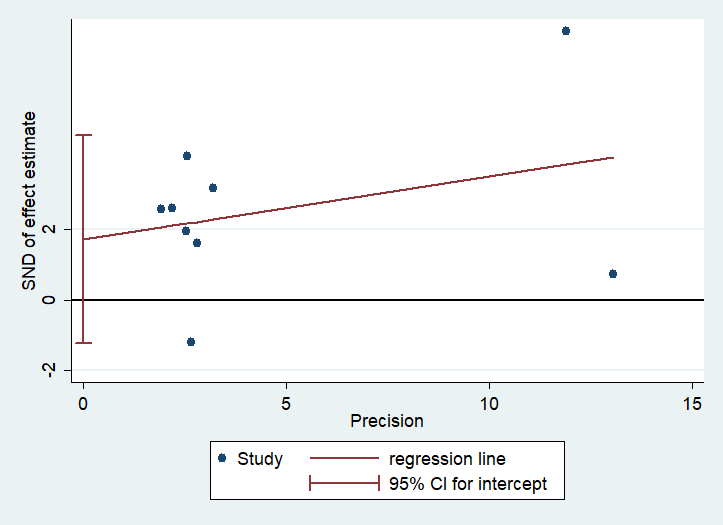


E) Tumor size


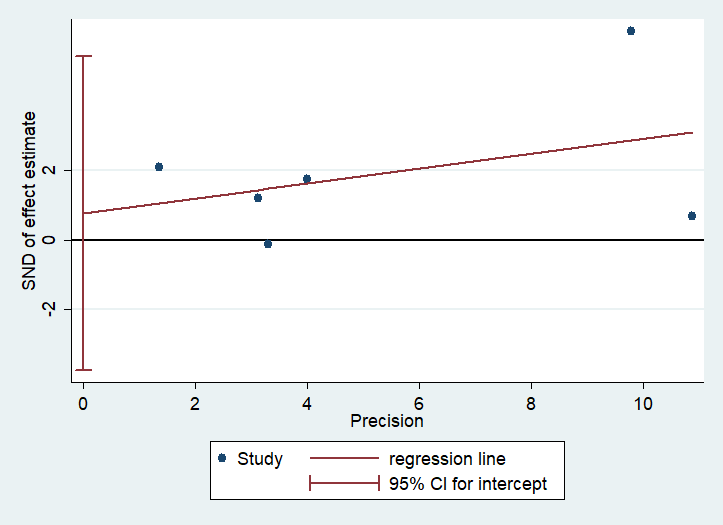


F) CLNM


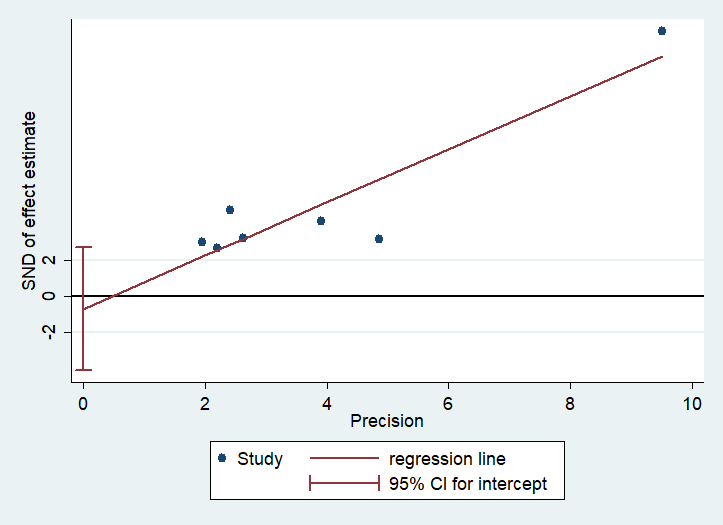


G) HT


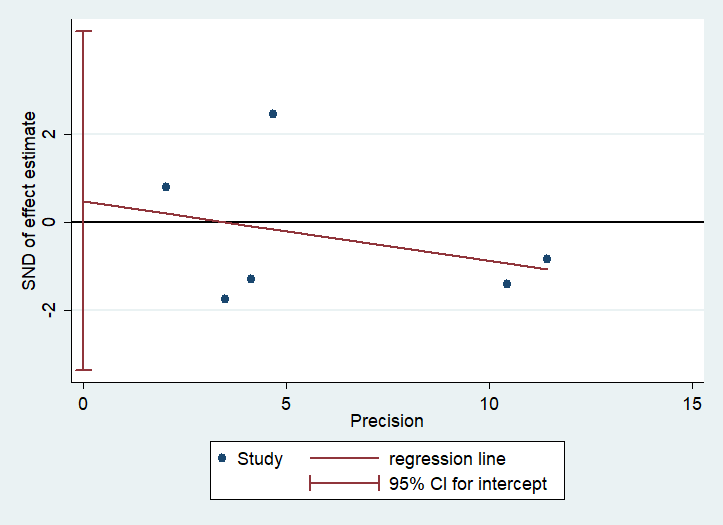


H) Contact>25%


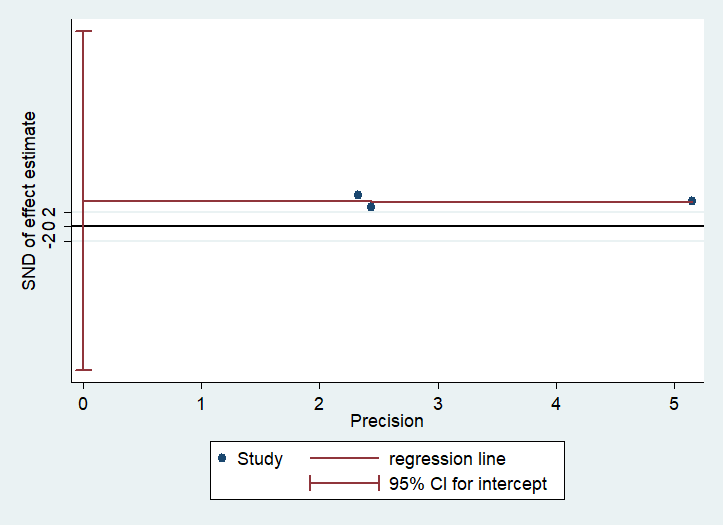


I) Calcification


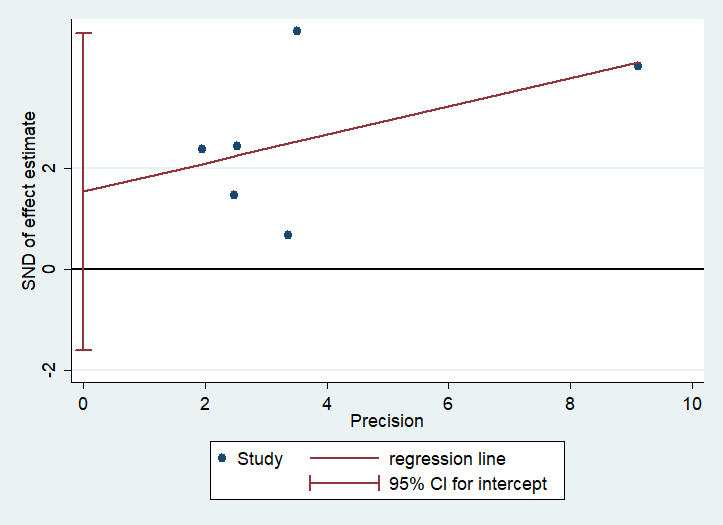


J) Composition


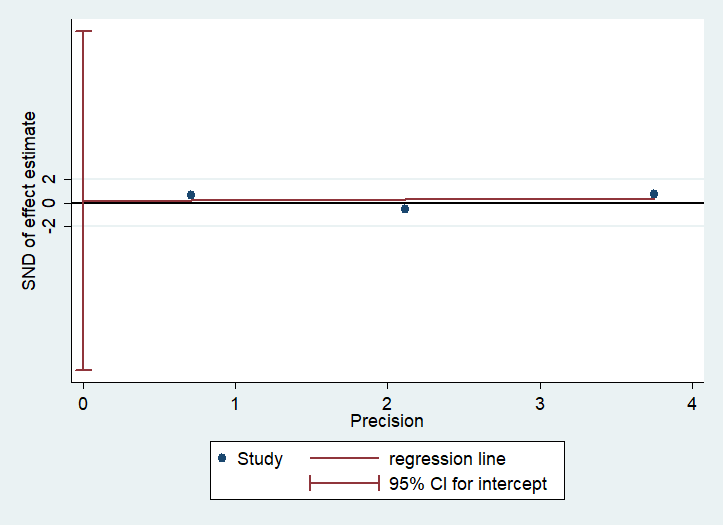


K) Echo


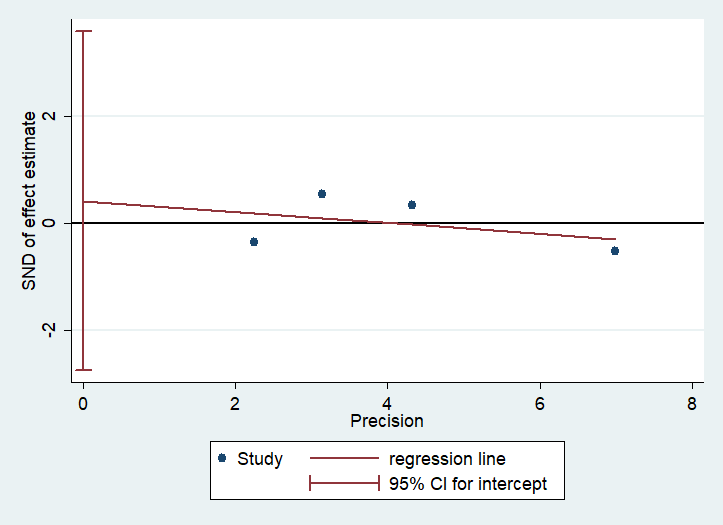


L) Margin


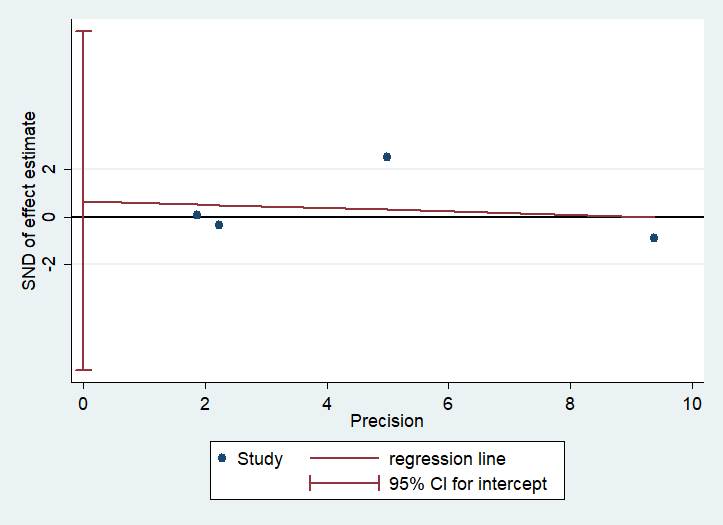


M) Shape


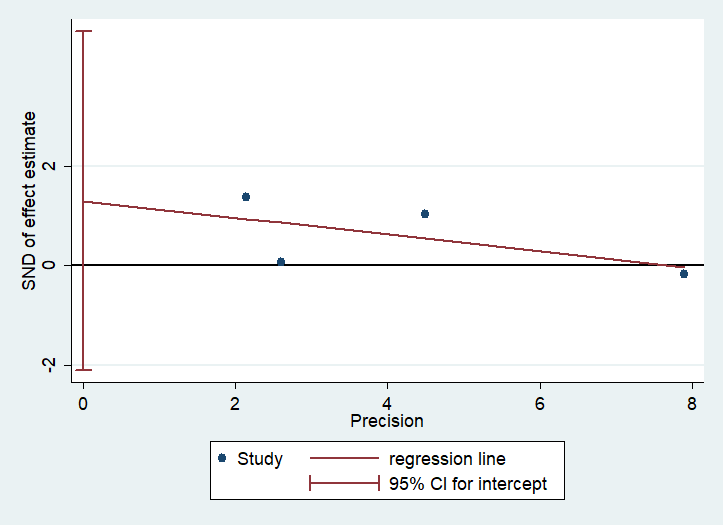


N) Location


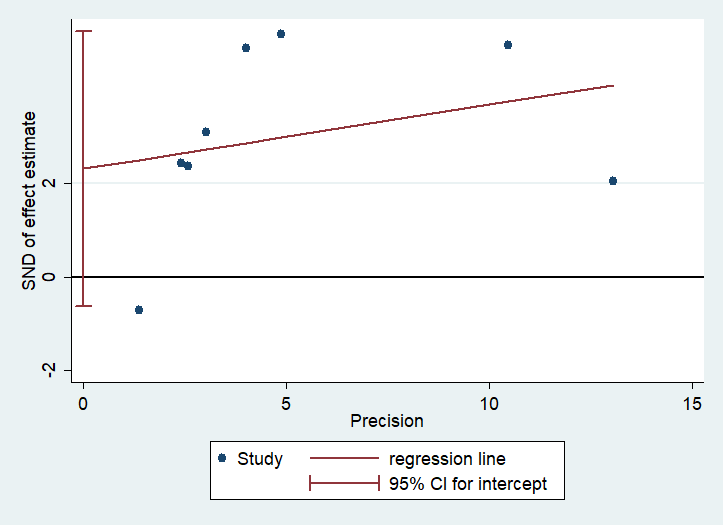

Supplement: Supplementary file 1 [file Data_Sheet_1.zip › S3 file.DOCX]
