# Supplementary material for: Clinical and Ultrasonic Risk Factors for Lateral Lymph Node Metastasis in Papillary Thyroid Microcarcinoma: A Systematic Review and Meta-Analysis
Source: Front Oncol. 2020 Apr 3;10:436. doi: 10.3389/fonc.2020.00436 (PMC7145902; doi:10.3389/fonc.2020.00436)
Supplement: Supplementary file 1 [file Data_Sheet_1.zip › S1 file.DOC]

Pubmed

#1 lymph node [Mesh] lateral[tiab] 955

#2 (lateral lymph node metastasis) OR (lateral neck dissection) OR (lateral lymph node dissection) OR (lateral neck metastasis) OR (Dissection, Lateral Neck) OR (Dissection, Lateral lymph node) 3511

#3 #1 OR #2 3744

#4 Thyroid Cancer, Papillary [MeSH Terms] microcarcinoma[tiab] 117

#5 "Papillary Thyroid Microcarcinoma"[Supplementary Concept] OR "Papillary Thyroid Microcarcinoma"[Tiab] OR "papillary thyroid microcarcinoma"[Tiab] 632

#6 #4 OR #5 672

#7 #3 and #6 78

Embase

#1 **'lymph node'**/exp AND **lateral**:ti,ab,kw 2776

#2 'lateral lymph node metastasis'/exp OR 'lateral lymph node metastasis' OR 'lateral neck dissection'/exp OR 'lateral neck dissection' OR 'lateral lymph node dissection' OR 'lateral neck metastasis' OR 'dissection, lateral neck' OR 'dissection, lateral lymph node)' 843

#3 #1 or #2 3389

#4 'papillary thyroid carcinoma'/exp AND lateral:ti,ab,kw 507

#5 'papillary thyroid microcarcinoma':ti,ab,kw 786

#6 #4 or #5 1227

#7 #3 and #6 291

Web of Science

#1 TOPIC: (lateral lymph node metastasis) OR TOPIC: (lateral neck dissection) OR TOPIC: (lateral lymph node dissection) OR TOPIC: (lateral neck metastasis) 2653

#2 TOPIC: (papillary thyroid microcarcinoma) 1547

#3 #1 and #2 192

Cochrane Library

#1 Mesh descriptor: [lymph nodes] explode trees 757

#2 lateral: ti,ab,kw 13410

#3 #1 and #2 20

#4 (lateral lymph node metastasis) OR (lateral neck dissection) OR (lateral lymph node dissection) OR (lateral neck metastasis) OR (Dissection, Lateral Neck) OR (Dissection, Lateral lymph node) 488

#5 #3 or #4 212

#6 Mesh descriptor: [thyroid cancer, papillary] explode trees 26

#7 microcarcinoma:ti,ab,kw 21

#8 #6 and #7 0

#9 (papillary thyroid microcarcinoma):ti,ab,kw 22

#10 #8 or #9 22

#11 #5 and #10 0
