# Supplementary material for: Clinical and Ultrasonic Risk Factors for Lateral Lymph Node Metastasis in Papillary Thyroid Microcarcinoma: A Systematic Review and Meta-Analysis
Source: Front Oncol. 2020 Apr 3;10:436. doi: 10.3389/fonc.2020.00436 (PMC7145902; doi:10.3389/fonc.2020.00436)
Supplement: Supplementary file 1 [file Data_Sheet_1.zip › S2 file.DOC]

Table S2 Sensitivity analysis summary

| Variables | P | I^2^ | Removed study | P | I^2^ | Possible reason |
| --- | --- | --- | --- | --- | --- | --- |
| age | 0.05 | 52 | Liu Z 2017 | 0.51 | 0 | Categorize 45-yr patients into younger subgroup |
| sex | 0.009 | 56 | Lin DZ 2016 | 0.62 | 0 | Not consecutive patients |
| ETE | 0.00 | 90 | Not found |  |  | ETE different diagnostic criteria (capsule; soft tissue or gross) |
| multifocal | 0.005 | 63 | Liu Z 2017 | 0.08 | 45 | Patients selection bias |
| HT | 0.07 | 50 | Zeng RC 2012 | 0.49 | 0 | Different diagnostic criteria |
| contact>25% | 0.02 | 75 | Kwak JY 2009 | 0.51 | 0 | Different US criteria or processor-dependent discrepancy |
| calcification | 0.01 | 65 | Zeng RC 2012 | 0.19 | 35 | Different US criteria or processor-dependent discrepancy |
| margin | 0.10 | 52 | Zeng RC 2012 | 0.92 | 0 | Different US criteria or processor-dependent discrepancy |
| location | 0.09 | 44 | Not found |  |  | Different US criteria or processor-dependent discrepancy |

ETE: extrathyroidal extension; HT: Hashimoto’s thyroiditis; US: ultrasound;

Figure S2: Forest plots for sensitivity analysis

A) Age

B) Sex

C)ETE

C) Multifocal

D) HT

E) Contact>25%

F) Calcification

G) Margin

H) Location
